# Postoperative C-reactive protein/albumin ratio as a novel predictor for short-term complications following gastrectomy of gastric cancer

**DOI:** 10.1186/s12957-017-1258-5

**Published:** 2017-10-24

**Authors:** Feng Sun, Xiaolong Ge, Zhijian Liu, Shangce Du, Shichao Ai, Wenxian Guan

**Affiliations:** 1 Department of General Surgery, Drum Tower Hospital, Medical School of Nanjing University, Nanjing, Jiangsu 210008 China; 2 Department of General Surgery, Sir Run Run Shaw Hospital, School of Medicine, Zhejiang University, Hangzhou, Zhejiang 310016 China

**Keywords:** C-reactive protein to albumin ratio, Postoperative complications, Gastric cancer

## Abstract

**Background:**

Postoperative complications following gastric cancer resection remain a clinical problem. Early detection of postoperative complications is needed before critical illness develops. The purpose of this study was to evaluate the prognostic value of C-reactive protein/albumin ratio in patients with gastric cancer.

**Methods:**

A total of 322 patients undergoing curative (R0) gastrectomy between 2015 and 2017 were retrospectively analyzed. Univariate and multivariate analyses were performed to identify clinical factors predicting postoperative complications. The cutoff values and diagnostic accuracy of C-reactive protein/albumin ratio and C-reactive protein were determined by receiver-operating characteristic curves.

**Results:**

Among all of the patients, 85 (26.4%) developed postoperative complications. The optimal cutoff of C-reactive protein/albumin ratio was set at 3.04 based on the ROC analysis. Multivariate analysis identified C-reactive protein/albumin ratio was an independent risk factors for complications after gastrectomy (OR 3.037; 95% CI 1.248–7.392; *P* = 0.014). Additionally, C-reactive protein/albumin ratio showed a higher diagnostic accuracy than C-reactive protein on postoperative day 3 (AUC: 0.685 vs 0.660; sensitivity: 0.624 vs 0.471; specificity: 0.722 vs 0.835).

**Conclusions:**

Elevated C-reactive protein/albumin ratio was an independent predictor for postoperative complications following gastrectomy of gastric cancer, and the diagnostic accuracy was higher than C-reactive protein alone. Overall, postoperative C-reactive protein/albumin ratio may help to identify patients with high probability of postoperative complications.

## Background

There are almost 1,000,000 new cases of gastric cancer every year worldwide, and half of these occur in Eastern Asia, particularly in China. Although the incidence of gastric cancer has declined over the years, it remains the fifth most common cancer and the third leading cause of cancer-related death in the world [1]. So far, surgical resection is still the primary treatment for resectable gastric cancer. Concomitantly, gastrectomy for gastric cancer may lead to high rates of postoperative complication, which has a negative effect on hospital recovery and long-term survival [2–5]. So, it is necessary to identify accurate predictive factors to predict postoperative complications early after surgery.

Several systemic inflammatory markers, including the Glasgow Prognostic Score (GPS), C-reactive protein (CRP), platelet to lymphocyte ratio (PLR), and neutrophil to lymphocyte ratio (NLR), have been established to predict postoperative complication [6]. As an acute-phase protein, CRP was widely studied in large number of surgery [7, 8]. Recently, a few studies began studying the predictive value of CRP for gastric cancer resection [9, 10]. In addition, predictors showing nutritional status, such as hypoalbuminemia, low body mass index (BMI), and weight loss, were also reported to be associated with postoperative recovery of gastric cancer [11, 12].

As a combination of these two aspects, C-reactive protein/albumin ratio (CAR) has been shown to be a promising prognostic index in pancreatic cancer [13], colorectal cancer, and renal cell cancer et al. [14, 15]. Liu et al. and Toiyama et al. have reported, respectively, that elevated CAR was related to a poor prognosis for gastric cancer resection [16, 17]. The former studies have largely been focused on preoperative CAR but to a lesser extent on that after surgery. Until now, whether altered postoperative CAR is associated with poor prognosis remains unclear. In this study, we evaluated the predictive value of postoperative CAR for short-term complications after gastric cancer resection.

## Methods

### Patients

A total of 322 patients who underwent curative (R0) gastrectomy between October 2015 and March 2017 in Nanjing Drum Tower Hospital were retrospectively analyzed. All of the patients were histologically confirmed, and blood laboratory tests on postoperative day (POD) 3 were complete. The exclusion criteria were as follows: (1) patients accepting ALB infusion preoperatively or within POD 3; (2) patients with liver cirrhosis and infection before gastrectomy which may have an influence on the serum albumin level; (3) reoperation within POD 3; (4) multivisceral resection. This study was approved by the Ethics Committee of Drum Tower Hospital, Medial School of Nanjing University.

### Data extraction

Data extraction included three aspects: the baseline characteristics, laboratory tests, and intraoperative index. The baseline characteristics were collected, including age, gender, BMI, comorbidities, and American Society of Anesthesiologists (ASA) grade. Blood laboratory tests included preoperative serum albumin, hemoglobin, CRP; postoperative CRP, albumin on POD 3; tumor markers [carbohydrate antigen 19–9 (CA19–9), carcinoembryonic antigen (CEA)]. Intraoperative index involved operation time, surgical approach, degree of lymph node dissection, and blood loss. Tumor stage was based on the 7th Edition of TNM Classification for Gastric Cancer [18].

### Definition of postoperative complications

The postoperative complications were termed as those occurred in hospital or within 30 days after surgery. All complications were categorized based on the Clavien-Dindo classification system [19]. Patients with postoperative complications of grades I or II were divided into minor group, while patients with complications of grades III or more were divided into major group. Besides postoperative short-term complications, the length of hospital stay was also collected to evaluate outcomes of patients with surgery. The CRP/Alb ratio was calculated as serum CRP level to serum ALB level both on POD 3 [14, 20]. CRP and CAR cutoff threshold were both determined based on receiver operating characteristic (ROC) curve analysis.

### Statistical analysis

Continuous variable data were presented as means ± SE and analyzed using Student’s *t* test or Mann-Whitney *U* test. Categorical variable data were presented as number (%) and analyzed using the Chi squared test or the fisher exact test. Univariate and multivariate analyses were performed to evaluate risk factors for the early postoperative complications. Accuracy of each optional risk factor was measured using receiver operating characteristic analysis. All of the statistical analyses were performed using SPSS 19.0 (Chicago, IL, USA), and statistical differences were termed as *P* < 0.05.

## Results

### Patient characteristics

Of the total of 322 patients, 232 were male while 90 were female. Among these patients with gastric cancer, 300 (93.2%) underwent open gastrectomy and 22 (6.8%) underwent laparoscopic surgery. Total gastrectomy was performed in 170 (52.8%) patients; distal gastrectomy and proximal gastrectomy were, respectively, in 114 (35.4%) and 38(11.8%). According to the 7th Edition of TNM Classification for Gastric Cancer, the number of patients with stage I/II/III/IV were 101/58/148/15 respectively. The mean operation time was 236.7 ± 65.7 min; mean blood loss was 229.6 ± 145.5 ml; mean length of postoperative stay was 15.4 ± 7.8 days. Overall, 85 (26.4%) patients had postoperative complications. Of those, 56 (17.4%) patients had minor complications [10 fever (> 38.5 °C) after surgery, 5 dumping syndrome, 17 delayed gastric emptying, 2 intestinal pseudoobstruction, 11 wound infection, 3 anemia and 8 TPN > 2 weeks], while 29 (9.0%) had major complications (5 anastomotic leakage, 5 fascial dehiscence, 2 adhesive intestinal obstruction, 3 abdomino-pelvic collection, 8 pleural effusion, 3 lymphatic leakage, 2 intra-abdominal bleeding and 1 death) according to the Clavien-Dindo classification system. Detailed clinicopathologic characteristics of all patients were shown in Table 1.

**Table 1:** Demographic and clinical features of patients

Characteristic	*N* = 322	Characteristic	*N* = 322
Age (years)	62.3 ± 9.9	CRP on POD 3 (mg/L)	96.7 ± 59.5
Gender (*n*)		ALB on POD 3 (g/L)	32.9 ± 3.6
Male	232	ASA ≥ 3	171
Female	90	Clinical stage I/II/III/IV	101/58/148/15
BMI (kg/m^2^)	23.0 ± 3.3	Mode of surgical approach (*n*, %)	
Comorbidities (*n*)		Laparoscopic	22
Diabetes mellitus	20	Open	300
Hypertension	111	Type of resection (*n*, %)	
Preoperative serum albumin (g/L)	38.6 ± 3.2	Distal gastrectomy	114
Preoperative hemoglobin (g/L)	123.1 ± 25.2	Proximal gastrectomy	38
Preoperative CRP (g/L)	6.2 ± 10.4	Total gastrectomy	170
CA 19–9 (ng/ml)		Degree of lymph node dissection (D) ≥ 2	246
≥ 37	60	Operation time (min)	236.7 ± 65.7
< 37	262	Blood loss (ml)	229.6 ± 145.5
CEA (ng/ml)		Postoperative complications (Clavien-Dindo)	
≥ 5	39	Grades I and II	56
< 5	283	≥ Grade III	29
Lymphocyte count (×10^9^/L)		Postoperative stay (days)	15.4 ± 7.8
≥ 3	13		
< 3	309		

*BMI* body mass index, *CA 19–9* carbohydrate antigen 19–9, *CEA* carcinoembryonic antigen, *CRP* C-reactive protein, *POD* postoperative day, *ASA* American Society of Anesthesiologists, *ALB* albumin

### Association between clinicopathologic characteristics and postoperative complications

As shown in Table 2, univariate analysis revealed that postoperative complications were significantly associated with CRP on POD 3, postoperative CAR, and degree of lymph node dissection. In further multivariate analysis, unlike CRP on POD 3, postoperative CAR (OR 3.037; 95% CI 1.248–7.392; *P* = 0.014) was still significantly associated with postoperative complications. These data indicated that postoperative CAP might be an independent predictor for early postoperative complications.

**Table 2:** Univariate and multivariate analyses of risk factors associated with postoperative complications

	Univariate			Multivariate		
Characteristics	OR	95% CI	*P*	OR	95% CI	*P*
Age (≥ 75 years)	1.441	0.621–3.343	0.395	1.827	0.641–5.212	0.260
Sex	0.721	0.422–1.234	0.233	0.703	0.361–1.371	0.301
BMI (< 18.5 kg/m^2^)	0.384	0.085–1.725	0.212	0.269	0.050–1.447	0.126
Comorbidities						
Diabetes mellitus	2.433	0.971–6.096	0.058	2.310	0.784–6.812	0.129
Hypertension	0.628	0.364–1.085	0.095	0.717	0.364–1.411	0.335
Preoperative serum albumin (< 35 g/L)	0.745	0.326–1.701	0.485	0.274	0.092–0.810	0.019
Preoperative hemoglobin (< 120 g/L)	1.587	0.957–2.630	0.073	2.023	1.027–3.985	0.042
Preoperative CRP (≥ 10 g/L)	1.030	0.417–2.544	0.949	0.965	0.304–3.059	0.951
CA 19–9 (≥ 37 ng/ml)	1.376	0.747–2.536	0.306	1.363	0.615–3.020	0.446
CEA (≥ 5 ng/ml)	0.956	0.445–2.057	0.909	0.681	0.267–1.738	0.422
Lymphocyte count (≥ 3 × 10^9^/L)	0.223	0.029–1.743	0.153	0.164	0.019–1.447	0.104
CRP on POD 3 (mg/L)	4.513	2.611–7.799	<0.001	2.376	0.955–5.913	0.063
Postoperative CAR	4.291	2.544–7.237	<0.001	3.037	1.248–7.392	0.014
ASA ≥ 3	0.818	0.498–1.343	0.427	0.793	0.415–1.519	0.485
Clinical stage (≥ II)	1.010	0.590–1.729	0.971	0.565	0.271–1.177	0.128
Mode of surgical approach	0.809	0.289–2.264	0.686	0.787	0.228–2.715	0.704
Type of resection	0.945	0.576–1.553	0.824	0.858	0.452–1.630	0.641
Degree of lymph node dissection (D) ≥ 2	2.542	1.269–5.092	0.008	4.429	1.884–10.410	0.001
Operation time (≥ 250 min)	1.544	0.934–2.553	0.090	1.513	0.791–2.893	0.211
Blood loss (≥ 200 ml)	1.443	0.845–2.466	0.179	1.424	0.762–2.659	0.268

*BMI* body mass index, *CA 19–9* carbohydrate antigen 19–9, *CEA* Carcinoembryonic antigen, *CRP* C-reactive protein, *POD* Postoperative day, *ASA* American Society of Anesthesiologists, *CAR* CRP to albumin (CRP/ALB) ratio, *OR* odds ratio, *CI* confidence interval

### Predictive value of CAR for postoperative complications compared with CRP on POD 3

**Fig. 1:**
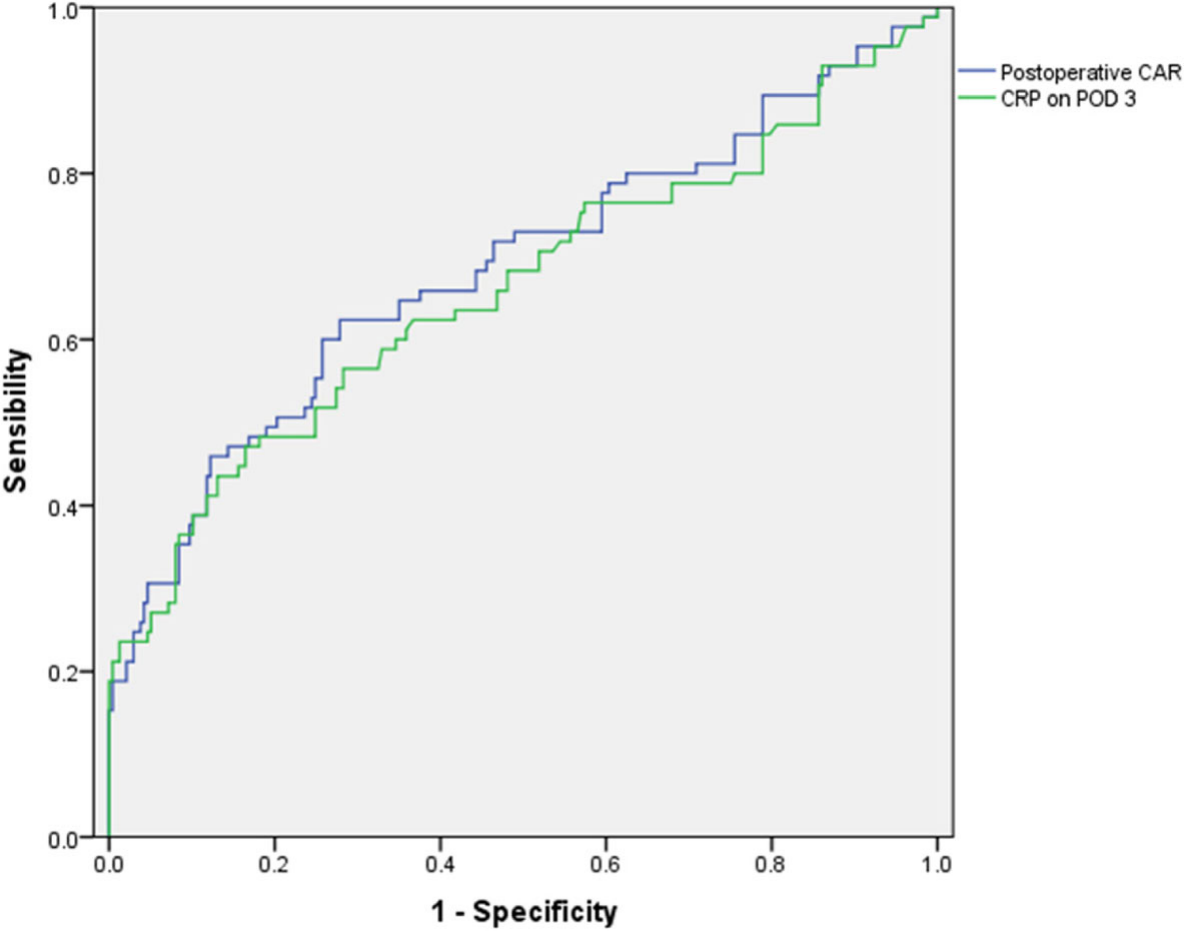
Receiver operating characteristic curve showing postoperative CAR and C-reactive protein on postoperative day 3 levels predictive of postoperative overall complications

Previous work demonstrated that postoperative CRP could be an optional predictor for complications after gastrectomy [9, 10]. To compare the predictive accuracy of postoperative CRP and postoperative CAR, receiver operating characteristic curve was performed. According to ROC analysis of all complications, the optimal cut-off value was 131.9 for CRP on POD 3, 3.04 for CAR respectively. ROC curve parameters were shown in Fig. 1. The AUC of CRP on POD 3 was 0.660, sensitivity was 0.471, specificity was 0.835, and Youden’s index was 0.306. In contrast, the AUC of CAR was 0.685, sensitivity was 0.624, specificity was 0.722, and Youden’s index was 0.345. All these data showed that postoperative CAR might be a better predictor for early postoperative complications than postoperative CRP.

### Postoperative CAR as a predictor for postoperative complications

Patients were divided into two groups: high CAR (≥ 3.04), low CAR (< 3.04). Patients with high CAR were more likely to have postoperative complications than those with low CAR (44.5 vs 15.8%, *P* < 0.001). Specifically, the possibility of suffering mild complications (grades I and II) and major complications (grades III or more) in patients with high CAR were both higher than those with low CAR (30.3 vs 9.9%, *p* < 0.001; 14.3 vs 5.9%, *P* = 0.011). In addition, patients with CAR also had prolonged hospital stay (17.1 ± 10.3 vs 14.5 ± 5.6, *P* = 0.003) (Table 3).

**Table 3:** Comparison of postoperative complications associated with postoperative CAR

Characteristics	All (*n* = 322)	CAR < 3.04 (*n* = 203)	CAR ≥ 3.04 (*n* = 119)	*P* value
Overall, *n* (%)^a, c^	85 (26.4)	32 (15.8%)	53 (44.5%)	< 0.001
Grades I or II, *n* (%)^a, c^	56 (17.4)	20 (9.9%)	36 (30.3%)	< 0.001
Grade III or greater, *n* (%)^a, c^	29 (9.0)	12 (5.9%)	17 (14.3%)	0.011
Postoperative stay (days)^b^	15.4 ± 7.8	14.5 ± 5.6	17.1 ± 10.3	0.003

*CAR* C-reactive protein to albumin ratio

^a^Clavien-Dindo’s classification of surgical complication

^b^Values are expressed as the mean ± SD

^c^Values are expressed as n (%)

## Discussion

In this study, patients with high postoperative CAR were more likely to have postoperative complications, and prolonged hospital stay. Additionally, postoperative CAR seemed to be more accurate to predict complications than postoperative CRP. Therefore, the postoperative CAR might be a promising predictor for postoperative complications after gastrectomy for gastric cancer.

Surgical resection is the main treatment for resectable gastric cancer. Although gastric cancer resection greatly prolongs the survival of patients, it brings some harmful effects. The overall morbidity of postoperative complications and mortality rates were reported to be 13 to 38 and 2 to 8.5%, respectively [21]. Postoperative complications, such as anastomotic leakage, and abdominal abscess have a negative effect on short-term surgical outcomes. Patients would have higher medical costs, and prolonged hospital stays. On the other hand, postoperative complications, especially infection complications, could cause prolonged inflammation which provides an appropriate microenvironment for recurrence of gastric cancer, and finally affect long-term survival [2, 5, 22]. Thus, no matter in terms of short-term or long-term outcomes, postoperative complications can bring catastrophic effects. So, it makes sense to find an accurate biochemical marker to predict postoperative complications in early stage.

Previous studies have proven that either preoperative or postoperative CRP could be an important predictive factor for both short-term outcomes and long-term mortality [10, 23–25]. As one of the inflammatory markers, CRP elevation mainly owes to the inflammatory reaction for cancer and surgical procedure. Shishido and colleagues evaluated that postoperative CRP on POD 3 could predict infectious complications after gastric cancer resection [10]. Kim et al. also demonstrated that postoperative CRP was a more accurate predictor for postoperative complications than other inflammatory markers, such as platelet count, neutrophil count, and ratios of these two factors [9]. Although postoperative CRP holds promise for prediction postoperative complications, there are still some limitations for being used widely in clinical practice. The main drawbacks of CRP are predictive accuracy and time lag [26, 27]. A recent study showed that the time point postoperative CRP began changing was late than some other inflammatory markers, like Interleukin-6 [26]. In our study, multivariate analysis also showed that postoperative CRP on POD 3 was not an independent risk factor for complications following gastrectomy.

To improve the accuracy of CRP, we modified this predictive index by introducing another factor: albumin. Albumin is produced in liver and accounts for the most abundant serum protein [28]. The serum albumin declines in patients with poor nutritional status, loss of skeletal muscle, and systemic inflammatory response [29]. The decline in albumin is due to the reprioritizes from visceral proteins to acute phase proteins happening in the liver, capillary leakage and hemodilution with fluid infusion [30, 31]. Furthermore, hypoalbuminemia has been proven to be a predictive marker for postoperative outcomes [32]. Ryan et al. revealed that postoperative hypoalbuminemia was associated with complications after esophagectomy [33]. Ge and his colleague also showed that the decrease of serum albumin could predict postoperative complications following colorectal resection [34]. In this study, we merged CRP and albumin together to calculate a single index: CRP/Alb ratio. Evaluation showed that postoperative CAR had higher AUC than postoperative CRP alone, and was an independent risk factor for postoperative complications following gastrectomy.

In this article, we chose the POD 3 as the time point for calculating CAR for several reasons. On one hand, there have been compelling suggestions that postoperative CRP reaches a peak at POD 3 or 4, and its predictive accuracy is better than that in POD 1 or 2 [9, 10]. On the other hand, the minimal albumin levels begin in 4–6 h after surgery and last for 3 days [27, 35]. Since both CRP and albumin own the maximal amplitude on POD 3, it would be reasonable to assume that CAR on POD 3 has the highest predictive accuracy.

To our knowledge, this is the first study to consider the postoperative CAR as a predictor for postoperative complications following gastrectomy for gastric cancer. Our results corresponded to previous studies in other kinds of cancer, such as hepatocellular cancer [36], lung cancer [37], and pancreatic cancer [13] et al. Although a good many research groups studied CAR, most of them focused on preoperative CAR. Considering inflammation from surgical trauma, postoperative CAR may be also a promising predictor for postoperative complications. Taken together, early detection of postoperative CAR may be beneficial to take action promptly before critical complications develop.

We acknowledge that there are some limitations in this study. First, the use of CAR on POD 3 may be a bit late for surgeons to perform preventive interventions. Second, it was a retrospective study, so it may be flawed by residual confounding factors. Third, patients enrolled in this study were from a single center. As the surgical technique and perioperative management played an important effect on postoperative complications, multicenter studies are needed to confirm our results. Lastly, the CAR cut-off value may be biased in this study for it was calculated using ROC analysis.

## Conclusions

In conclusion, the current study demonstrated that postoperative CAR is an independent predictive marker for short-term complications following gastrectomy of gastric cancer. Patients with high CAR (≥ 3.04) need to be treated seriously before critical complications develop.

## Abbreviations

ASA: American Society of Anesthesiologists
CA19–9: Carbohydrate antigen 19–9
CAR: C-reactive protein/albumin ratio
CEA: Carcinoembryonic antigen
CRP: C-reactive protein
POD: Postoperative day
ROC: Receiver-operating characteristic
